# A Systematic Way to Find Linear Motifs Mediating Protein–Protein Interactions

**DOI:** 10.1371/journal.pbio.0030424

**Published:** 2005-11-15

If John Donne had been a systems biologist, instead of writing “No man is an island,” he might have written “No protein is an island.” Proteins in cells interact with numerous partners, forming complex networks that keep cells ticking over, allow them to respond to external stimuli, and make sure that essential processes like cell division go smoothly.

Interacting proteins can be identified experimentally in many ways. For example, an antibody recognizing one protein can be used to pull that protein plus any proteins that are bound to it out of cell extracts. High-throughput assays have also been developed that can screen whole genomes for genes encoding interacting proteins. But we remain a long way from having an accurate picture of all the protein–protein interactions in even one cell, let alone a whole organism.

Proteins interact with each other in two main ways. The first is through globular domains, which are formed when linear strings of 100–200 amino acids fold into specific shapes determined by their amino acid sequence. The second is through a globular domain in one protein and a short linear sequence (motif) of three to eight amino acids in the other. The structures of globular domains are relatively easy to solve, and how they interact in well-established pairs of interacting proteins can be visualized. Furthermore, other potentially interacting domains can then be inferred through sequence similarity to known globular domains. By contrast, the linear motifs involved in protein–protein interactions can't be easily identified by sequence comparisons. Until now, the only way to find them has been through time-consuming experiments, and consequently, only a few hundred linear motifs are known.

Now, a systematic way to find linear motifs in the billions of sequences stored in databases, devised and tested by Victor Neduva et al., ushers in a new era in understanding protein–protein interaction networks. The researchers started with the hypothesis that a set of proteins with a common interacting partner will share a feature that mediates binding. This feature could be a domain or a linear motif. To find the latter, they stripped away the sequences of globular domains and of long repetitive regions to leave behind the nonstructured parts of the proteins, regions where they believe linear motifs are most likely to lie. They then determined whether any short sequences in these protein remnants occurred more frequently within the set of interacting proteins than would be expected by chance. These statistically significant short sequences are potential linear motifs involved in protein binding.

The researchers tested their approach on protein sets sharing known motifs, and showed that it efficiently identified these motifs while minimizing the number of false positives. They then extracted protein sets that shared a common interaction partner from four species-specific datasets of protein–protein interactions and ran their protocol for finding linear motifs. From a fly dataset, for example, they identified 26 protein sets with one or more linear motifs that occurred more frequently than expected by chance. That nine of these motifs were already known provided important validation of their approach, but the researchers also checked several of the new motifs in direct binding experiments. For example, they showed that a motif predicted to bind to the fly protein Translin did in fact bind to it; a mutated version of the motif did not.

From their results, Neduva et al. estimate that hundreds of linear motifs may remain to be discovered. Given the central role that linear motifs play in protein–protein interactions, their systematic method for finding them should rapidly improve our understanding of the complex network of protein–protein interactions that drives the everyday lives of cells. —*Jane Bradbury*

